# Adenylyl Cyclase (AC) Mediates the Antidepressant-Like Effects of Tropisetron on a Mouse Model of Maternal Separation Stress

**DOI:** 10.1155/2021/5586119

**Published:** 2021-04-22

**Authors:** Ali Hosseinzadeh, Shakiba Nasiri Boroujeni, Elham Saghaei, Zahra Loriooini, Saeid Habibian Dehkordi, Shima Balali-Dehkordi, Mohammad Rahimi-Madiseh, Hossein Amini-Khoei

**Affiliations:** ^1^Medical Plants Research Center, Basic Health Sciences Institute, Shahrekord University of Medical Sciences, Shahrekord, Iran; ^2^Department of Basic Sciences, Veterinary Faculty, Shahrekord University, Shahrekord, Iran

## Abstract

The adenylyl cyclase (AC) pathway is involved in the pathophysiology of depression. Finding new antidepressants with high medicinal properties and low side effects is warranted. Therefore, this study was designed to determine the antidepressant-like effect of tropisetron on a maternal separation (MS) model in mice, considering the possible role of AC. NMRI male mice were divided into eleven groups. The control group was treated with saline and MS groups were treated with saline, tropisetron (a 5-HT3 receptor antagonist) at doses of 1, 3, and 5 mg/kg; forskolin (an activator of AC) at doses of 5, 10, and 25 mg/kg; a subeffective dose of forskolin with a subeffective dose of tropisetron; and an effective dose of tropisetron plus an effective dose of NB001 (3 mg/kg) (an AC inhibitor). After treatment, animals were subjected to behavioral tests including the forced swimming test (FST), splash test, and open field test (OFT). We showed that MS caused depressive-like behaviors determined as an increase in the immobility time in the forced swimming test (FST) and decreased grooming time in the splash test. Our results showed that administration of tropisetron, as well as forskolin, mitigated the depressive-like behaviors in MS mice. We found that coadministration of a subeffective dose of tropisetron plus a subeffective dose of forskolin potentiated the antidepressant-like effect of tropisetron. However, coadministration of an effective dose of NB001 with an effective dose of tropisetron did not significantly affect the antidepressant-like effect of tropisetron. We concluded that the antidepressant-like effects of tropisetron on MS mice are partially mediated through the adenylyl cyclase pathway.

## 1. Introduction

Depression is one of the most common psychological disorders [1]. More than 15% of people experience at least one period of depression in developed countries in their lifetime [2, 3]. The emotional relationship between the mother and the infant in childhood has been shown to play an important role in neurodevelopment and behavioral responses in adulthood [4]. Maternal care at early life is associated with improved biological behavior and physiological development and subsequently increases social adjustment [5]. Experiencing unfortunate events like maternal separation (MS) in the early stages of life has a negative effect on behavior and brain development and potentially acts as a risk factor for psychological disorders such as depression in adulthood [6–8].

Monoamine serotonin or 5-hydroxytryptamine (5-HT) is an important neurotransmitter in the pathophysiology of depressive disorder and is also involved in the mechanisms of action of commonly used antidepressants [9]. Studies in both animal and clinical models have shown that depression is clearly associated with a decrease in the level of serotonin in the central nervous system (CNS) [10–13]. 5-HT regulates various biological functions such as mood, sleep, appetite, daily rhythms, and energy balance [14]. Evidence suggests that the 5-HT3 receptor, as ion channel ligands, is involved in brain development and maturation. This receptor is widely distributed in the CNS and plays an important role in regulating various processes and different brain functions. Previous studies have shown that the 5-HT3 receptor antagonists, including tropisetron and ondansetron, possessed antidepressant-like properties in animal models of depression [15, 16]. However, the exact mechanisms that are involved in the antidepressant-like effect of tropisetron have not been fully determined.

Adenylyl cyclase (AC) is an enzyme that converts ATP to cAMP [17, 18]. A number of studies have shown that AC is involved in the pathophysiology of depression [19, 20]. Platelet AC activity has been shown to act as a biological marker for the evaluation of the depressive state [21–24]. This is based on the fact that people with a history of depression have lower levels of platelet AC activity [25]. Forskolin is one of the AC agonists that increase the formation of intracellular cAMP. Previous studies have showed that forskolin can be considered as an agent with potential antidepressant effect [6, 8, 26, 27].

Considering that tropisetron exerted an antidepressant-like effect and also involvement of AC in the pathophysiology of depression, the present study is designed to evaluate the possible involvement of AC in the antidepressant-like effect of tropisetron on a mouse model of MS-induced depression.

## 2. Material and Methods

### 2.1. Maternal Separation (MS) Paradigm

Pregnant NMRI mice (Pasteur Institute, Tehran, Iran) were used. The animals were kept under standard laboratory conditions (12-hour light/dark cycle, 22 ± 1°C and free access to water and food). To perform the MS paradigm as described by Lorigooini et al., the birthday was considered as postnatal day 0 (PND0). Pups from PND2 to PND14 were then separated from their mothers for 3 hours a day and then returned to their mother's cage. At the end of PND14, the mice were returned to their mother's cage and remained there until PND21 [28]. On day 21, male mice were divided into ten groups (*n* = 8) until the day of the experiment (PND60). All procedures were carried out in accordance with the regulations of the university and the Guide for the Care and Use of Laboratory Animals of National Institutes of Health (ethics code: IR.SKUMS.REC.1397.120) and Guide for the Care and Use of Laboratory Animals (8th edition, National Academies Press). Full efforts were made to reduce the use of animals and to advance their welfare.

### 2.2. Experimental Groups

Mice were divided into 11 groups (*n* = 6) as follows: group 1—control mice which received normal saline; group 2—MS mice which received normal saline; groups 3–5—MS mice which received tropisetron at doses of 1, 3, and 5 mg/kg, respectively; groups 6–8—MS mice which received forskolin (an activator of AC) at doses of 5, 10, and 25 mg/kg, respectively; group 9—MS mice cotreated with tropisetron at a dose of 1 mg/kg (subeffective dose) plus a subeffective dose of forskolin (5 mg/kg), simultaneously; group 10—MS mice cotreated with an effective dose of tropisetron (5 mg/kg) plus an effective dose of AC inhibitor (NB001, 3 mg/kg), simultaneously; and group 11—MS mice which received fluoxetine at a dose of 5 mg/kg. The dose and time of drug administration were chosen based on previous studies and our pilot study [29–33]. Drugs were dissolved in physiological saline and injected as a single dose via an intraperitoneal (i.p.) route with a volume of 5 ml/kg body weight. Agents were injected one hour before the behavioral tests. 132 mice were used in this study. Different sets of mice were used for behavioral tests. One set for the OFT and FST and another set for the splash test.

### 2.3. Open Field Test (OFT)

The OFT was performed to evaluate the locomotion following treatments. The OFT was done immediately before the FST to consider ambulatory behavior as well as to confirm that adjustments which occur in motor activity did not affect the immobility time in the FST. The OFT device is a white Plexiglas with dimensions of 30 × 50 × 50 cm. Each mouse was gently placed at the center of the device. Its movements were recorded for 5 minutes by the camera and evaluated by EthoVision version 8 software. In the OFT, the horizontal (number of crossing by 4 foots from each square) activity was measured. The apparatus was cleaned with 70% ethanol after the experiment with each mouse [28, 34].

### 2.4. Forced Swimming Test (FST)

In this test, the immobility time of mice, along with frustrating behavior as a reflection of depressive-like behavior, was recorded. To do this, a glass container (25 × 12 × 15 cm) was filled with 25°C water and the mouse was placed gently in the water from a height of 20 cm. Disruption of mouse movements was considered immobility. The test time was 6 minutes; the first 2 minutes was considered to match the animal with the current conditions, and the immobility time was measured for the next 4 minutes [35].

### 2.5. Splash Test

The splash test was used to examine personal care and motivational problems in mice. To do this, a 10% sucrose solution was sprayed on the dorsal coat of the mouse and its behavior was filmed for 5 minutes. In this test, self-cleaning activities including nose/face cleaning, head washing, and body grooming were measured [36].

### 2.6. Data Analysis

Data were analysed using Prism software, and then, the results were displayed as mean ± S.E.M. Data were analysed using one-way ANOVA and Tukey's post hoc test. *P* < 0.05 was considered statistically significant.

## 3. Results

### 3.1. Locomotor Activity in the OFT

**Figure 1:**
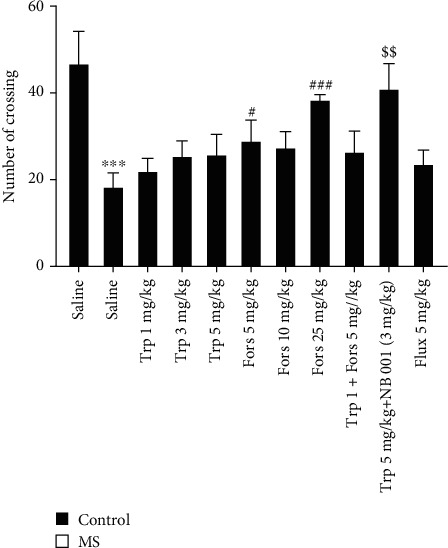
Horizontal activity in the OFT. Values are presented as the mean ± S.E.M. from 6 animals and were analysed using one-way ANOVA followed by Tukey's post test. Drugs were dissolved in physiological saline and injected as a single dose via intraperitoneal (i.p.) route with a volume of 5 ml/kg body weight one hour before the test. ^∗∗∗^*P* < 0.001 compared with the saline-treated control group, ^#^*P* < 0.05 and ^###^*P* < 0.001 compared with the saline-treated MS group, and ^$$^*P* < 0.01 compared with the tropisetron- (5 mg/kg) received MS group. MS: maternal separation; Trp: tropisetron; Flux: fluoxetine; Fors: forskolin.

One-way ANOVA analysis showed that there are significant differences amongst experimental groups in the case of horizontal activity (*F*(10, 55) = 16.46, *P* < 0.001). Findings showed that the MS group's horizontal activities were significantly lower than those of the control group (*P* < 0.001, Figure 1). The horizontal activities in the MS mice who received forskolin at doses of 5 and 25 mg/kg were significantly higher than those in the saline-treated MS group (*P* > 0.05 and *P* < 0.001, respectively). We observed that the horizontal activities in MS mice that received coadministration of tropisetron (5 mg/kg) plus NB001 were significantly increased compared to those in the group that received tropisetron at the dose of 5 mg/kg alone (*P* < 0.01). We did not observe significant alterations in cases of horizontal activities in groups that received tropisetron at different doses as well as fluoxetine in comparison with those in saline-treated control mice (*P* > 0.05).

### 3.2. Immobility Time in the FST

**Figure 2:**
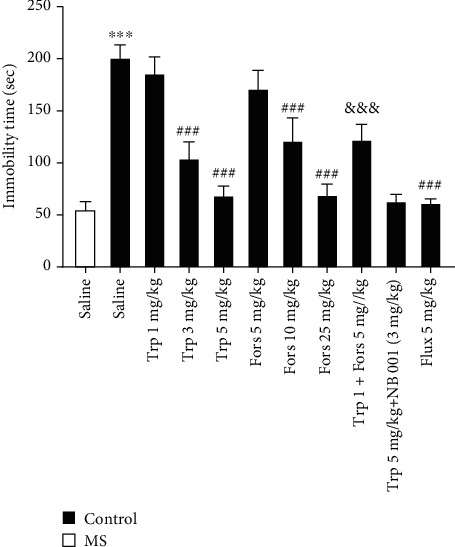
Immobility time in the FST. Values are presented as the mean ± S.E.M. from 6 animals and were analysed using one-way ANOVA followed by Tukey's post test. Drugs were dissolved in physiological saline and injected as a single dose via intraperitoneal (i.p.) route with a volume of 5 ml/kg body weight one hour before the test. ^∗∗∗^*P* < 0.001 compared with the saline-treated control group, ^###^*P* < 0.001 compared with the saline-treated MS group, and ^&&&^*P* < 0.001 compared with the tropisetron- (1 mg/kg) received MS group. MS: maternal separation; Trp: tropisetron; Flux: fluoxetine; Fors: forskolin.

One-way ANOVA analysis showed that there are significant differences amongst experimental groups in cases of immobility time in the FST (*F* (10, 55) = 56.18, *P* < 0.001). As shown in Figure 2, the duration of immobility in the FST in the MS group was significantly longer than that in the control group (*P* < 0.001). The immobility time in the MS groups that received tropisetron at the doses of 3 and 5 mg/kg was significantly reduced compared to that in the saline-treated MS mice (*P* < 0.001). The forskolin-received MS mice (doses of 10 and 25 mg/kg) have lower immobility time compared to the saline-treated MS group (*P* < 0.001). Coadministration of subeffective doses of tropisetron (1 mg/kg) with a subeffective dose of forskolin (5 mg/kg) significantly reduced the immobilization time compared to that of the MS group that received the subeffective dose of tropisetron alone (*P* < 0.001). In addition, coadministration of effective doses of tropisetron (5 mg/kg) with an effective dose of NB001 (3 mg/kg) did not significantly reduce the immobility time compared to that of the MS group that received the effective dose of tropisetron alone (*P* > 0.05). Results demonstrated that fluoxetine (5 mg/kg) significantly decreased the immobility time in comparison with that of saline-treated MS mice (*P* < 0.001).

### 3.3. Grooming Activity Time in the Splash Test

**Figure 3:**
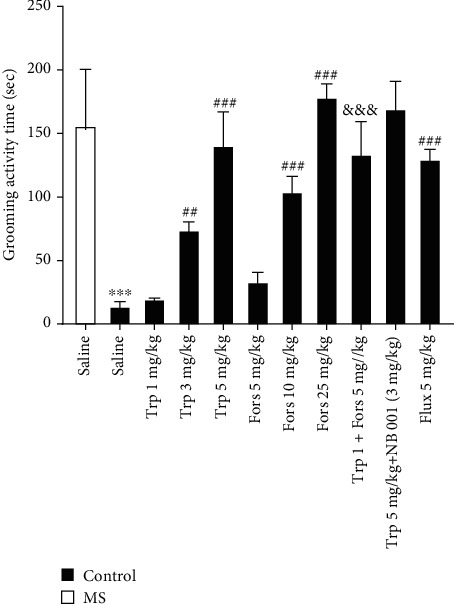
Grooming activity time in the splash test. Values are presented as the mean ± S.E.M. from 6 animals and were analysed using one-way ANOVA followed by Tukey's post test. Drugs were dissolved in physiological saline and injected as a single dose via intraperitoneal (i.p.) route with a volume of 5 ml/kg body weight one hour before the test. ^∗∗∗^*P* < 0.001 compared with the saline-treated control group, ^##^*P* < 0.01 and ^###^*P* < 0.001 compared with the saline-treated MS group, and ^&&&^*P* < 0.001 compared with the tropisetron- (1 mg/kg) received MS group. MS: maternal separation; Trp: tropisetron; Flux: fluoxetine; Fors: forskolin.

One-way ANOVA analysis determined that the grooming activity time is significantly different amongst experimental groups (*F* (10, 55) = 36.24, *P* < 0.001). According to the results (Figure 3), the grooming activity time in the splash test in the MS group was significantly lower than that in the control group (*P* < 0.001). Administration of tropisetron at doses of 3 and 5 mg/kg significantly increased the grooming activity time in the MS mice compared to the saline-treated MS mice (*P* < 0.01 and *P* < 0.001, respectively). Our results showed that the grooming activity time in the MS groups that received forskolin at doses of 10 and 25 mg/kg significantly increased compared to those in the saline-received MS counterparts (*P* < 0.001). Simultaneous administration of subeffective doses of tropisetron (1 mg/kg) plus a subeffective dose of forskolin (5 mg/kg) significantly increased the grooming activity time compared to that of the MS group that received a subeffective dose of tropisetron alone (*P* < 0.001). Furthermore, coadministration of effective doses of tropisetron (5 mg/kg) plus an effective dose of NB001 (3 mg/kg) did not significantly increase the grooming activity time compared to that of the MS group that received the effective dose of tropisetron alone (*P* > 0.05). Results showed that fluoxetine (5 mg/kg) significantly increased the grooming activity time in comparison with that of saline-treated MS mice (*P* < 0.001).

## 4. Discussion

Results of the present study showed that MS provoked depressive-like behaviors in the FST and splash test as the increase in the immobility time and the decrease in the grooming activity time, respectively. We showed that tropisetron mitigated the negative effect of MS on mouse behaviors. Findings showed that forskolin (an activator of AC) potentiated the antidepressant-like effect of the subeffective dose of tropisetron.

Previous studies have shown that MS increases the risk of depressive-like behaviors in adulthood [37]. It has been determined that MS changes the neurotransmitter systems, such as the serotonergic system in the brain, and creates long-term and enduring negative effects on brain development and behavior in the adulthood [38]. Disruption in the serotonergic system is strongly involved in the pathophysiology of depression [39]. Ample evidence showed that MS increased the duration of immobility in the FST and reduced the grooming activity time in the splash test [37]. Our findings are in agreement with previous studies and determined that the MS causes depressive-like behaviors in the FST and splash test, as immobility time in MS mice increased in the FST test and the self-care and self-cleaning times significantly decreased in the splash test. In addition, we performed the OFT to approve that locomotor activity subsequent of treatments does not affect the FST results and the immobility of animals in the FST is not associated with their hypolocomotion [40]. The OFT was performed directly before the FST to measure ambulatory behavior and approve that inconsistencies which happen in motor activity did not affect the immobility time in the FST.

In 2006, Bravo and Maswood showed that treatment with a single dose of tropisetron reduced the rate of immobility in the FST [35]. In 2016, Haj-Mirzaian et al. conducted a study examining the contribution of nitric oxide and guanosine monophosphate cycles to the antidepressant-like effect of tropisetron and ondansetron on mice. They found that nitric oxide mediated the antidepressant-like effect of 5-HT3 antagonists in the FST and tail suspension test [41]. In 2016, Kordjazy et al. conducted a study and determined that NMDA receptors are involved in the antidepressant-like effect of 5-HT3 antagonists [15]. In 2016, Haj-Mirzaian et al. demonstrated that tropisetron significantly reduced the immobility in the FST and increased grooming activity in the splash test in mice [42]. The results of the mentioned studies confirm the results obtained from our study. In which, tropisetron exerted antidepressant effects in the FST, OFT, and splash test. However, the exact and full underlying mechanisms that are involved in the antidepressant-like effect of tropisetron have not been understood. Thus, in this study, we evaluated the possible involvement of the adenylyl cyclase pathway in this beneficial effect of tropisetron.

In 1996, Menninger and Tabakoff conducted a study to evaluate the effect of forskolin as a stimulant of platelet adenylyl cyclase in people with depression. This study indicates that the difference in the activity of platelet adenylyl cyclase is related to the difference in people with a history of depression [43]. Lowther et al., in 1996, showed that the AC receptor had a lower level of stimulation in depressed patients [44]. In 2005, Hines and Tabakoff conducted a study to evaluate the activity of platelet AC as a marker in depression. Their results showed that people with depression have lower levels of platelet AC activity [22]. The results of these studies confirm the effective role of AC in the development of depressive symptoms. The results of the present study are in line with the results of the above studies. We showed that stimulation of the AC receptor using forskolin potentiated the antidepressant-like effect of tropisetron. Surprisingly, we observed that inhibition of AC using NB001 did not attenuate the antidepressant-like effect of tropisetron. Further studies are needed to identify the reason for this response.

Bard et al. showed that stimulation of the 5-HT receptor in a dose-dependent manner causes transient accumulation of cAMP in cells which is similar to the activity of AC [45]. To date, no studies have investigated the role of the AC receptor in the antidepressant effect of tropisetron. The results of the present study showed that the subeffective dose of tropisetron in combination with the subeffective dose of the activator of AC (forskolin) enhanced the antidepressant-like effect of tropisetron in the FST and splash test. However, coadministration of tropisetron with the AC inhibitor (NB001) did not mitigate the beneficial effect of tropisetron. Our findings indicate that the AC receptor is involved in the antidepressant-like effect of tropisetron. It seems necessary to conduct more detailed studies in this field to clarify more this involvement.

## 5. Conclusion

The results of the present study showed that the administration of tropisetron in a dose-dependent manner exerted the antidepressant-like effect on MS mice. We found that the adenylyl cyclase receptor partially, at least, mediated the antidepressant-like effect of tropisetron.

## Data Availability

The original data used to support the findings of this study are included within the article.

## Additional Points

*Limitations of the Study*. The limitation of the study included a single and relatively short treatment period.
